# Dermatological conditions in intensive care: a secondary analysis of the Intensive Care National Audit & Research Centre (ICNARC) Case Mix Programme Database

**DOI:** 10.1186/cc6141

**Published:** 2008-01-18

**Authors:** Susannah MC George, David A Harrison, Catherine A Welch, Kathleen M Nolan, Peter S Friedmann

**Affiliations:** 1 Clinical Teaching Fellow in Specialties (Dermatology), St Helier Hospital, Wrythe Lane, Carshalton, Surrey SM5 1AA, UK; 2 Intensive Care National Audit & Research Centre, Tavistock House, Tavistock Square, London WC1H 9HR, UK; 3 Critical Care Directorate, Southampton General Hospital, Tremona Road, Southampton SO16 6YD, UK; 4 University of Southampton, Dermatopharmacology Unit, Southampton General Hospital, Tremona Road, Southampton SO16 6YD, UK

## Abstract

**Introduction:**

Dermatology is usually thought of as an outpatient specialty with low mortality, however some skin conditions require intensive care. These conditions are relatively rare and hence are best studied using clinical databases or disease registries. We interrogated a large, high-quality clinical database from a national audit of adult intensive care units (ICUs), with the aim of identifying and characterising patients with dermatological conditions requiring admission to ICU.

**Methods:**

Data were extracted for 476,224 admissions to 178 ICUs in England, Wales and Northern Ireland participating in the Case Mix Programme over the time period December 1995 to September 2006. We identified admissions with dermatological conditions from the primary and secondary reasons for admission to ICU.

**Results:**

A total of 2,245 dermatological admissions were identified. Conditions included infectious conditions (e.g. cutaneous cellulitis, necrotising fasciitis), dermatological malignancies, and acute skin failure (e.g. toxic epidermal necrolysis, Stevens–Johnson syndrome and autoimmune blistering diseases). These represent 0.47% of all ICU admissions, or approximately 2.1 dermatological admissions per ICU per year. Overall mortality was 28.1% in the ICU and 40.0% in hospital. Length of stay in intensive care was longest for those with acute skin failure (median 4.7 days for ICU survivors and 5.1 days for ICU non-survivors).

**Conclusion:**

We have identified patients who not only require intensive care, but also dermatological care. Such patients have high mortality rates and long ICU stays within the spectrum of the UK ICU population, similar to other acute medical conditions. This highlights the importance of skin failure as a distinct entity comparable to other organ system failures.

## Introduction

Dermatology is usually thought of as an outpatient specialty with low mortality. However, some skin conditions can be very severe and the condition itself, or associated complications of the condition or treatment, may cause the patient to require intensive care. There is little literature dealing specifically with intensive care dermatology [1,2]. Green *et al*. described the skin problems that may arise in a critically ill patient [1]. Dunnill *et al*. reported a case series of 27 dermatology patients admitted to a 30-bedded intensive care department at St Thomas's Hospital over a 14-month period [2].

Conditions that may require intensive care management include toxic epidermal necrolysis (TEN), Stevens–Johnson syndrome (SJS), necrotising fasciitis and exfoliative dermatitis. Patients with large areas of skin involvement have problems with fluid and electrolyte balance and with temperature control as a result of large daily percutaneous fluid losses [2]. In critically ill patients, hypoalbuminaemia is typically caused by the systemic inflammatory response syndrome and redistribution. However, in patients with extensive skin loss, hypoalbuminaemia also occurs as a result of cutaneous losses, hypercatabolic state and decreased synthesis [2]. Patients with impaired barrier function are also vulnerable to infection. In 1991, Irvine defined the concept of skin failure and proposed that it was a real entity comparable to any other major organ system dysfunction [3]. However, the concept of skin failure is not well known [4], and skin conditions that may require intensive care are relatively rare. For example, the incidence of TEN is reported to be between 0.4 and 1.3 cases per million person years and that of SJS between 1–7 cases per million person years (which equate to approximately 50 and 240 cases per year, respectively, in the UK) [5,6]. The incidence of necrotising fasciitis is estimated at 500 new cases per year in the UK [7]. This makes it difficult for individual clinicians to gain experience of managing such cases. Conditions with such a low incidence are best studied using large high quality clinical databases arising from multicentre audits or disease registries [8-10]. The collective experience of managing these rare cases can then be collated.

Our aim was to describe the frequency, physiological parameters, length of stay, and mortality of patients with dermatological conditions admitted to UK intensive care units (ICUs) participating in a national audit of intensive care over a 10-year period.

## Materials and methods

### The Case Mix Programme Database

The Case Mix Programme (CMP) is the national, comparative audit of adult, general critical care units (ICUs and combined intensive care and high dependency units) in England, Wales and Northern Ireland. Data are extracted by trained data collectors and undergo extensive local and central validation prior to inclusion in the CMP Database [11]. Validated data for all admissions to units participating in the CMP between December 1995 and September 2006 were extracted from the CMP Database.

The CMP has received approval from the Patient Information Advisory Group (PIAG), under Section 60 of the Health and Social Care Act 2001, for the collection and use of patient identifiable information without individual patient consent (approval no. PIAG 2-10(f)/2005).

### Selection of cases

In the CMP, reasons for admission to the ICU are recorded using a hierarchical coding method, the Intensive Care National Audit & Research Centre (ICNARC) coding method [12]. Cases were selected if the primary or secondary reason for their admission to ICU was recorded as any of the following conditions, coded within the dermatological system of the ICNARC coding method:

• necrotising fasciitis;

• cutaneous cellulitis;

• orbital cellulitis;

• cutaneous melanoma;

• basal cell carcinoma;

• toxic epidermal necrolysis;

• Stevens–Johnson syndrome;

• erythema multiforme;

• exfoliative dermatitis;

• pemphigus vulgaris;

• psoriasis and pustular psoriasis;

• scleroderma.

If both the primary and secondary reasons for admission were dermatological, then the patient was categorised according to the primary reason for admission. In addition, patients were identified if their primary or secondary reason for admission was partially coded within the dermatological system of the hierarchical coding method. Partial codes are used when a specific condition cannot be identified within the coding method. Partially-coded reasons were reclassified, where possible, using the process tier of the coding method (e.g. infection or malignancy) and the free text field.

### Analyses

An analysis plan was agreed *a priori*. For each of the individual conditions listed above, we summarised the number of admissions and the numbers of these dying in the admitting ICU, transferred to another ICU, and dying before ultimate discharge from an acute hospital.

The following three groups of conditions (as classified in Table 1) were analysed separately and in more detail:

**Table 1:** Dermatological conditions in the Case Mix Programme Database

Condition	ICU admissions, n (%*)	ICU deaths, n (%)	ICU transfers, n (%)	Ultimate hospital deaths, n (%)
Infective conditions:				
Necrotising fasciitis	1,133 (0.24)	336 (29.7)	56 (4.9)	438 (41.6)
Cutaneous cellulitis	658 (0.14)	193 (29.3)	14 (2.1)	274 (42.6)
Orbital cellulitis	48 (0.01)	8 (16.7)	1 (2.1)	12 (26.7)
Wound infection†	28 (0.01)	2 (7.1)	0 (0.0)	10 (35.7)
Infected ulcer†	27 (0.01)	10 (37.0)	0 (0.0)	17 (65.4)
Abscess†	23 (< 0.01)	2 (8.7)	1 (4.3)	4 (18.2)
Gangrene†	6 (< 0.01)	1 (16.7)	0 (0.0)	1 (16.7)
Infected eczema†	3 (< 0.01)	1 (33.3)	0 (0.0)	1 (33.3)
Dermatological malignancies:				
Cutaneous melanoma	80 (0.02)	6 (7.5)	0 (0.0)	13 (16.9)
Basal cell carcinoma	96 (0.02)	6 (6.3)	0 (0.0)	11 (12.0)
Squamous cell carcinoma†	15 (< 0.01)	0 (0.0)	0 (0.0)	0 (0.0)
Acute skin failure:				
Toxic epidermal necrolysis	86 (0.02)	32 (37.2)	5 (5.8)	43 (50.6)
Stevens–Johnson syndrome	46 (0.01)	14 (30.4)	5 (10.9)	19 (46.3)
Erythema multiforme	19 (< 0.01)	3 (15.8)	2 (10.5)	6 (31.6)
Psoriasis and pustular psoriasis	19 (< 0.01)	8 (42.1)	0 (0.0)	8 (42.1)
Exfoliative dermatitis	16 (< 0.01)	7 (43.8)	0 (0.0)	7 (43.8)
Pemphigus vulgaris	9 (< 0.01)	4 (44.4)	0 (0.0)	4 (44.4)
Cutaneous T cell lymphoma†	2 (< 0.01)	1 (50.0)	0 (0.0)	2 (100.0)
Staphylococcal scalded skin syndrome†	1 (< 0.01)	0 (0.0)	0 (0.0)	0 (0.0)
Epidermolysis bullosa†	1 (< 0.01)	1 (100.0)	0 (0.0)	1 (100.0)
Other:				
Scleroderma	36 (0.01)	15 (41.7)	1 (2.8)	19 (54.3)
Rash due to systemic infection†	18 (< 0.01)	3 (16.7)	1 (5.6)	5 (38.5)
Pressure sores†	10 (< 0.01)	2 (20.0)	0 (0.0)	3 (33.3)
Allergic reaction†	7 (< 0.01)	0 (0.0)	0 (0.0)	0 (0.0)
Allergy testing†	5 (< 0.01)	0 (0.0)	0 (0.0)	0 (0.0)
Complication following skin surgery for non malignant condition†	4 (< 0.01)	1 (25.0)	0 (0.0)	1 (25.0)
Dermatomyositis†	2 (< 0.01)	1 (50.0)	0 (0.0)	2 (100.0)
Pyoderma gangrenosum†	2 (< 0.01)	2 (100.0)	0 (0.0)	2 (100.0)
Weber-Christian disease†	1 (< 0.01)	0 (0.0)	0 (0.0)	1 (100.0)
Behçet's syndrome†	1 (< 0.01)	0 (0.0)	0 (0.0)	0 (0.0)
Wegener's granulomatosis†	1 (< 0.01)	1 (100.0)	0 (0.0)	1 (100.0)
Hypereosinophilia†	1 (< 0.01)	1 (100.0)	0 (0.0)	1 (100.0)
Radiation necrosis†	1 (< 0.01)	0 (0.0)	0 (0.0)	0 (0.0)
Axillary hyperhidrosis (thoracic sympathectomy)†	1 (< 0.01)	0 (0.0)	0 (0.0)	0 (0.0)

Total	2,406 (0.51)	661 (27.5)	86 (3.6)	906 (39.6)

*Percentage of all admissions in the Case Mix Programme Database. †Identified from partial code and text field. ICU, intensive care unit.

1. Infective conditions;

2. Dermatological malignancies;

3. Conditions causing acute skin failure.

For these three subgroups, the following measures of case mix, activity and outcome were summarised.

#### Case mix

The case mix of admissions was described by the age, sex, surgical status, severe conditions in the past medical history, the Acute Physiology And Chronic Health Evaluation (APACHE) II score and predicted mortality [13], and the ICNARC physiology score and predicted mortality [14]. In addition, for admissions with TEN and related conditions (SJS and erythema multiforme), the disease-specific SCORTEN severity scoring system was also used. The SCORTEN comprises a score for seven factors relating to mortality: age greater than 40 years; heart rate greater than 120 min^-1^; cancer; involved body area greater than 10%; serum urea greater than 10 mmol.l^-1^; serum bicarbonate less than 20 mmol.l^-1^; and serum glucose greater than 14 mmol.l^-1 ^[15]. The APACHE II, ICNARC and SCORTEN models were calculated using computer algorithms based on raw data recorded from the first 24 h in ICU.

The most frequently occurring non-dermatological reasons for admission recorded in the database were also reported for all patients with dermatological conditions.

#### Outcome

Outcome was described by the mortality in the original admitting ICU and at ultimate discharge from an acute hospital. For admissions with TEN and related conditions, hospital mortality was also reported by SCORTEN. The ability of SCORTEN to discriminate between hospital survivors and non-survivors was evaluated by the area under the receiver operating characteristic (ROC) curve [16] and compared with the discrimination of the APACHE II score and the ICNARC risk prediction model.

The ROC curve is a plot of the sensitivity against the specificity of the score for predicting death at each different value. The area under the curve, also called the concordance or c-index, is equivalent to the probability that a randomly selected non-survivor will have a higher score than a randomly selected survivor.

#### Activity

Activity was described by the number of patients transferred to another ICU, the number transferred for more specialised care, the length of stay in ICU by ICU discharge status (discharged, transferred or died), and the total length of stay in an acute hospital by ultimate hospital survival status.

## Results

Out of 476,224 admissions to 178 adult general ICUs from 1 December 1995 to 30 September 2006, 2,245 were cases in which a dermatological condition was fully specified as the primary or secondary reason for admission. A further 213 partially coded dermatological reasons were identified. Of these, three could be given a full code within the coding method, 158 were categorised based on the free text field and the remaining 52 had insufficient detail recorded and were excluded from the analyses. The resulting 2,406 dermatological admissions (Table 1) represented 0.51% of all admissions in the database, or approximately two dermatological admissions per unit per year.

Overall, 661 patients (27.5%) with a dermatological condition died in intensive care with a total of 906 patients (39.6%) dying before ultimate discharge from an acute hospital. There were 86 (3.6%) transfers of patients to another ICU.

The most frequent dermatological reasons for admission to intensive care were infective conditions. There were 1,133 cases of necrotising fasciitis and 658 cases of cutaneous cellulitis, which together made up three quarters of all dermatological admissions to intensive care.

Excluding conditions representing fewer than 20 admissions, mortality was lowest for patients with cutaneous malignancies, with 11 (12.0%) hospital deaths amongst patients with basal cell carcinomas and highest for those with scleroderma with 19 (54.3%) hospital deaths.

Patients with dermatological malignancies were older than those with infective conditions or acute skin failure (mean age 65 years versus 58 and 52 years, respectively), and there was a higher percentage of male patients in this group (59% versus 52% and 45%) (Table 2).

**Table 2:** Case mix, outcome and activity for major subgroups of dermatological admissions

	Infective conditions	Dermatological malignancies	Acute skin failure
Admissions, n (% of all admissions)	1,926 (0.40)	191 (0.04)	199 (0.04)

Age, mean (SD)	57.6 (16.5)	65.1 (15.0)	51.7 (20.8)

Male, n (%)	1,009 (52.4)	112 (58.6)	90 (45.2)

Surgical status, n (%):			
Non-surgical	1,016 (52.8)	38 (19.9)	170 (85.4)
Elective	172 (8.9)	138 (72.3)	16 (8.0)
Emergency	737 (38.3)	15 (7.9)	13 (6.5)

Past medical history*, n (%):			
Steroid treatment	65 (3.4)	3 (1.6)	12 (6.1)
Chemotherapy	43 (2.3)	8 (4.2)	13 (6.6)
Metastatic disease	17 (0.9)	25 (13.2)	0 (0.0)
Chronic renal replacement therapy	37 (1.9)	1 (0.5)	5 (2.5)
Severe respiratory disease	32 (1.7)	1 (0.5)	3 (1.5)
Very severe cardiovascular disease	26 (1.4)	3 (1.6)	3 (1.5)
Radiotherapy	15 (0.8)	11 (5.8)	1 (0.5)
Lymphoma	19 (1.0)	1 (0.5)	6 (3.0)
Acute myelogenous/lymphocytic leukaemia or multiply myeloma	11 (0.6)	0 (0.0)	3 (1.5)
Portal hypertension	12 (0.6)	0 (0.0)	1 (0.5)
Biopsy proven cirrhosis	12 (0.6)	0 (0.0)	0 (0.0)
Chronic myelogenous/lymphocytic leukaemia	8 (0.4)	1 (0.5)	1 (0.5)
Home ventilation	8 (0.4)	1 (0.5)	0 (0.0)
Congenital immunohumoral or cellular immune deficiency state	5 (0.3)	0 (0.0)	1 (0.5)
Hepatic encephalopathy	5 (0.3)	0 (0.0)	0 (0.0)
AIDS	2 (0.1)	0 (0.0)	0 (0.0)

APACHE II score, mean (SD)	19.1 (7.5)	14.5 (5.3)	18.1 (7.0)

ICNARC model:			
Physiology score, mean (SD)	22.7 (10.4)	12.1 (6.7)	21.2 (10.6)
Predicted mortality, median (IQR)	37.6 (16.7–64.5)	10.1 (6.2–19.2)	32.7 (12.3–62.7)

ICU length of stay (days), median (IQR):			
ICU survivor	4.1 (1.7–10.0)	1.0 (0.8–2.0)	4.7 (1.9–14.8)
ICU non-survivor	1.9 (0.7–5.8)	1.0 (0.3–4.0)	4.7 (2.0–10.0)
Transfer to another ICU	4.0 (0.9–9.6)	-	3.5 (0.7–11.6)

Total acute hospital length of stay (days), median (IQR):			
Hospital survivor	39 (22–66)	17 (8–28)	34 (16–75)
Hospital non-survivor	9 (3–29)	6 (3–22)	15 (7–31)

ICU bed days (% of total bed days)	13,808 (0.60)	391 (0.02)	1,923 (0.08)

Transfer to another ICU, n (%)	72 (3.7)	0 (0.0)	12 (6.0)

ICU mortality, n (%)	553 (28.7)	12 (6.3)	70 (35.2)

Ultimate hospital mortality, n (%)	757 (41.5)	24 (13.0)	90 (46.6)

Values are n (%), mean (SD) or median (IQR), as indicated. *Percentage of admissions with evidence to assess past medical history. APACHE, Acute Physiology And Chronic Health Evaluation; ICNARC, Intensive Care National Audit & Research Centre; ICU, intensive care unit; IQR, interquartile range; SD, standard deviation.

Patients with infective conditions were mostly medical admissions (52.8%) or were admitted following emergency surgery (38.3%). By comparison, the vast majority of those with dermatological malignancies were admitted following elective surgery (72.3%) and most of those with acute skin failure were non-surgical admissions (85.4%) (Table 2).

Disease severity scores, APACHE II and the ICNARC physiology score, were highest in those with infective conditions and lowest in those with cutaneous malignancies.

Length of intensive care stay was longest for those with acute skin failure (median 4.7 days for both ICU survivors and non-survivors). Length of intensive care stay and total hospital stay was shortest for those with dermatological malignancies.

The most commonly recorded non-dermatological reasons for admission among these patients were for septic shock/septicaemia (*n *= 573, 23.8%), acute renal failure (*n *= 168, 7.0%) and pneumonia (*n *= 95, 4.0%) (Table 3).

**Table 3:** Top 10 most common non-dermatological reasons for admission for patients with both dermatological and non-dermatological reasons recorded

Reason	Associated non-dermatological reason for admission	n (%)
1	Septic shock/septicaemia	573 (23.8)
2	Acute renal failure	168 (7.0)
3	Pneumonia	95 (4.0)
4	Chronic obstructive pulmonary disease (COPD)	36 (1.5)
5	Non-cardiogenic pulmonary oedema	31 (1.3)
6	Diabetes mellitus	27 (1.1)
7	Supra-ventricular tachycardia, atrial fibrillation or flutter	26 (1.1)
8(=)	Hypovolaemic shock	25 (1.1)
8(=)	Morbid obesity	25 (1.1)
10	Chronic renal failure	22 (0.9)

**Figure 1:**
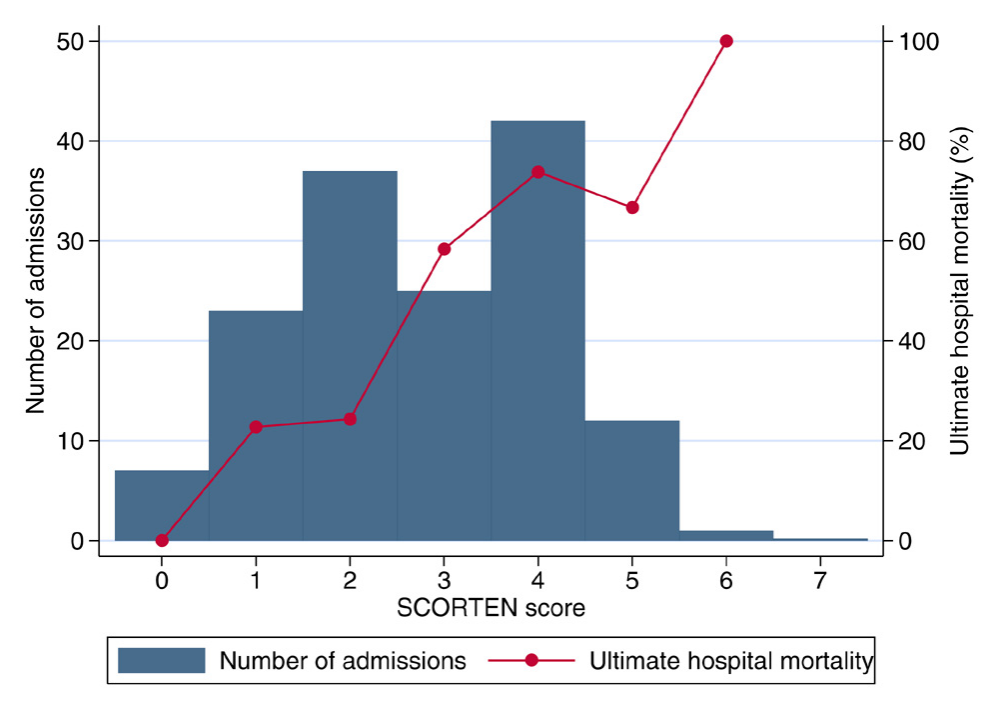
Ultimate hospital mortality by SCORTEN for admissions with toxic epidermal necrolysis and related conditions (*n *= 145).

**Figure 2:**
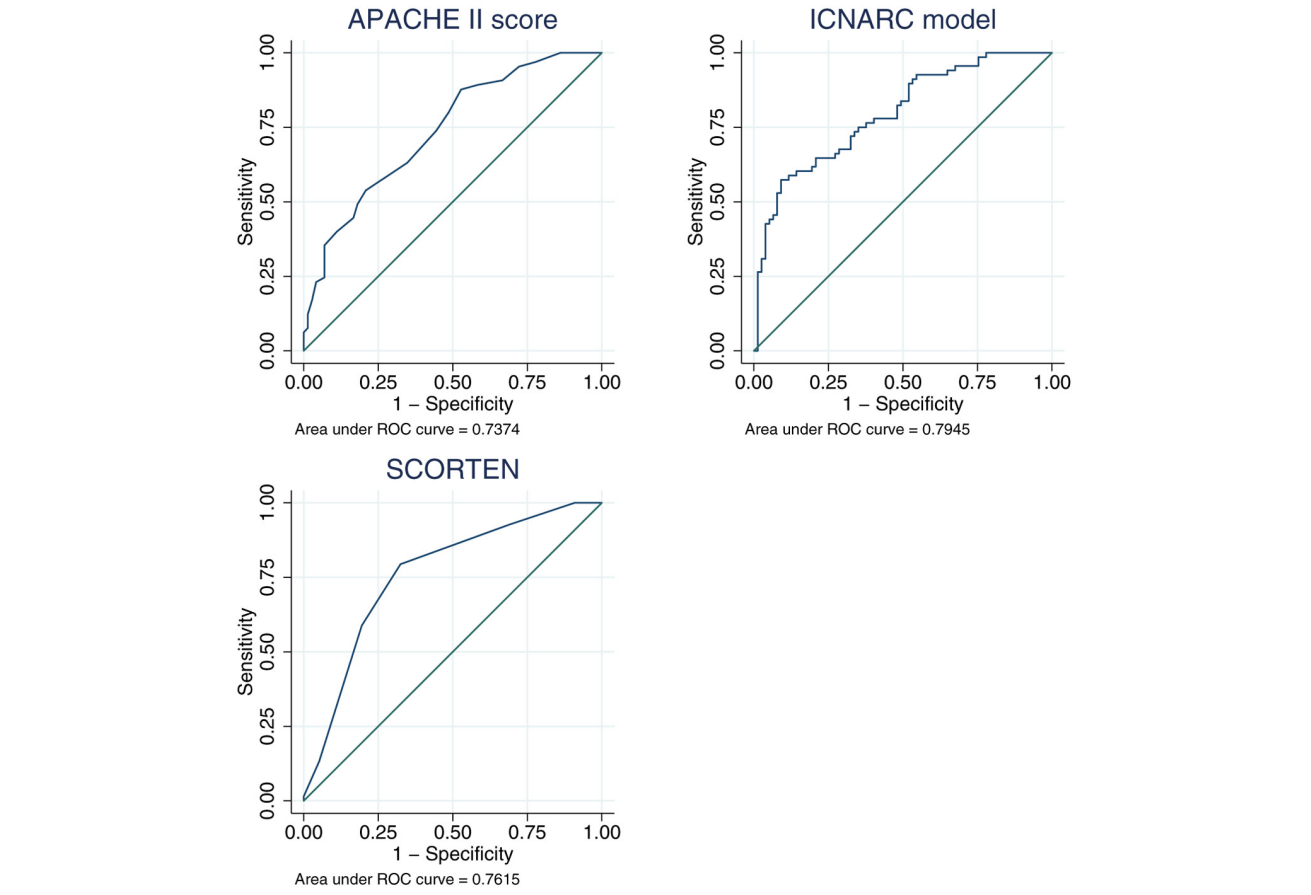
Receiver operating characteristic curves for SCORTEN, the APACHE II score and the ICNARC model for admissions with toxic epidermal necrolysis and related conditions (*n *= 145).

For admissions with TEN, SJS and erythema multiforme (*n *= 145), mortality increased steeply with SCORTEN (Figure 1), rising from around 20% for scores of 0–2 to over 70% for scores of 4 or more. The area under the ROC curve for SCORTEN was 0.762 (95% confidence interval 0.685–0.838) (Figure 2). This compared with values of 0.737 (0.655–0.819) for the APACHE II score (in 137 eligible patients), and 0.795 (0.722–0.867) for the ICNARC model, suggesting that the ICNARC model was best for discriminating between survivors and non-survivors in this patient group. However, with relatively small numbers, none of the differences in ROC curves were statistically significant (SCORTEN versus APACHE II, Chi-squared = 0.14, p = 0.73; SCORTEN versus ICNARC model, Chi-squared = 1.08, p = 0.30; ICNARC model versus APACHE II, Chi-squared = 1.52, p = 0.22).

## Discussion

This report provides information about the range of dermatological conditions requiring intensive care in England over an 11-year period (1995–2006). Of 476,224 admissions to 178 ICUs in England, Wales and Northern Ireland, 2,406 dermatological admissions were identified. While rare, these conditions are important as they have a high mortality and often require long intensive care and hospital stays. Overall, 28% of patients died in intensive care, with a total of 40% dying in hospital. Patients with acute skin failure had the highest mortality with 35% dying in intensive care and a total of 47% dying in hospital. By comparison, of the overall general adult intensive care population, 20% die in the ICU and 31% die in hospital [11]. While the population of patients admitted to intensive care is very heterogeneous, comparisons with the average for all intensive care admissions allow us to place the dermatological admissions within the spectrum of all ICU admissions. The hospital mortality of patients admitted to ICU with acute skin failure is comparable to that of other acute medical conditions, such as pancreatitis (42%) [17] and pneumonia (49%) [18].

As well as having higher mortality, dermatology patients with infective conditions and acute skin failure also have longer intensive care and hospital stays than average for the general adult intensive care population. The median length of stay in intensive care for all adult patients is 1.7 days for survivors and 2.0 days for non-survivors, whereas for dermatology patients, survivors with infective conditions and acute skin failure had median ICU stays of between 4 and 5 days. In the present study, ICU deaths in the acute skin failure group occurred particularly late, with a median ICU stay of 5 days for non-survivors. The median total hospital stay for patients admitted to ICUs is 16 days for survivors and 9 days for non-survivors [11]. Our results on hospital length of stay are consistent with two previous retrospective cohort studies of patients with necrotising fasciitis, which reported mean hospital stays of around 31 days for survivors and 12 days for non-survivors [19,20]. One of these studies also reported the overall mean ICU stay as 21 days [20].

Compared with the average for the general adult intensive care population, APACHE II scores were higher for dermatology patients with infective conditions and acute skin failure (mean scores 19.2 and 18.0, respectively), but lower for dermatological malignancies (mean score 14.5). These reflect typical scores for conditions that are predominantly emergency and elective, respectively. They compare with mean APACHE II scores of 16.5 for all UK intensive care patients [11] and 19.2 for a previous cohort of 166 patients with necrotising fasciitis [19].

The most common dermatological reason for admission to intensive care (1133 cases) was necrotising fasciitis. Over a 10-year period this corresponds to an overall treated incidence of approximately 280 patients per year in the UK. As the total UK incidence is estimated at 500 new cases per year [7], a significant proportion of these cases must be managed either in more specialised units, or more likely in high dependency units or on the general ward. Mortality in previously reported case series of patients with necrotising soft tissue infections varies from 17% to 33% [19-22]. Our patients with necrotising fasciitis had an overall mortality of 41.6%. This high mortality is most likely to be due to selection of only the most severe cases for treatment in an ICU due to the limited critical care resources in the UK.

Our study included 145 patients who had a diagnosis of TEN and related conditions (SJS and erythema multiforme). The data available in the CMP Database allowed us to produce ROC curves for SCORTEN, the APACHE II score and the ICNARC model for these cases. SCORTEN was developed using a sample of 165 patients and validated on a further 75 patients from the same dermatology ICU [15]. The area under the ROC curve in the validation sample was 0.82 (95% confidence interval 0.74 to 0.90), which indicates good discriminatory power. Further work using a large proportion of the patients from the original study found that SCORTEN calculated on any of the first 5 days of admission produced ROC values of over 0.8 [23]. The area under the ROC curve for SCORTEN in our study was 0.763, which was better than the APACHE II score (0.737) but not as good as the ICNARC model (0.795), although these differences were not statistically significant. SCORTEN is a simple model, which is quick and easy to use at the bedside. By comparison, APACHE II and the ICNARC model are more complex and are designed to give estimates of the risk of hospital mortality across all admissions to intensive care. The comparable performance of the simple SCORTEN model to these more complex models was therefore impressive.

Only a small number of patients had exfoliative dermatitis, pemphigus vulgaris, psoriasis and scleroderma, however all of these conditions had a high mortality. Even smaller numbers were identified with dermatomyositis, cutaneous T cell lymphoma, staphylococcal scalded skin syndrome and epidermolysis bullosa. The rarity of these conditions makes it difficult to gain experience of managing such cases.

The admission to ICU of patients with comparatively low risk conditions, such as basal cell carcinoma, may seem surprising. However, reviewing the free text field of the database for these cases, where used, indicated that these were likely to be admissions following complex surgery, in particular operations involving facial reconstruction. This would explain the relatively low but not insignificant mortality of 12% among this group.

Our study shows the most common non-dermatological reasons for admission in patients with both dermatological and non-dermatological reasons recorded were sepsis, acute renal failure and pneumonia. Unsurprisingly, these represent common and direct complications of severe skin disorders. They suggest that in some cases the skin condition alone does not precipitate admission to ICU, but rather the associated complications. In terms of pre-existing conditions, only the most severe illnesses in a patient's past medical history are routinely recorded in the CMP Database. A review of necrotising fasciitis found that pre-existing conditions included diabetes mellitus (incidence 21–64%), peripheral vascular disease (15–80%), intravenous drug abuse (8–77%), obesity (18–46%), chronic alcoholism (12–31%) and malnutrition (18–40%) [24]. More recent studies have found that the most frequent comorbidities were diabetes (19–37%) [19-21], obesity (17–31%) [20,21], hypertension (35%) [20] and intravenous drug abuse (30%) [19]. The low rates of conditions such as diabetes and obesity in our results reflect the fact that these conditions are only recorded in the CMP Database if they are considered to have had a significant influence on the decision to admit the patient to intensive care.

The dermatological admissions identified therefore represent a combination of those admitted for their primary dermatological condition, such as those with acute skin failure, a direct complication of that condition, such as septic shock or renal failure, or as a result of their treatment, such as those admitted for monitoring following major surgery or following an acute complication during minor surgery. It is in the first of these three groups where the involvement of dermatologists may be of particular benefit in terms of diagnostic advice and knowledge of therapeutic options.

The high mortality and long duration of ICU stay of patients with skin conditions compared to the general adult ICU population highlights the importance of skin failure as a cause of morbidity and mortality. Although conditions causing skin failure are rare, it is important to recognise that this is no less serious than any other organ system failure. As the broad aims of ICU management are to maintain homeostasis, disruption of skin function results in specific management problems. These necessitate a multidisciplinary approach to management with involvement of both intensivists and dermatologists, and meticulous nursing care. The recent UK government proposal to move dermatology services into the community [25] may reduce the availability of a specialist dermatological opinion and potentially compromise the care of this small number of severely ill patients.

The size of the dataset available provides information on a large number of patients; however it also has limitations in terms of lacking detail. For example, we have no information on the accuracy of the diagnoses, whether they were made by an intensivist or a dermatologist, and whether diagnosis was confirmed by skin biopsy. Assigning a diagnosis to such patients is further complicated by the various diagnostic criteria that may have been used, for example in cases of SJS or TEN [5]. In order to calculate SCORTEN we assumed that patients coded as TEN had involvement of greater than 10% of body surface area and other patients did not. Patients with between 10 and 30% body surface area involvement may be considered 'overlap TEN/SJS' [6] and, if these were recorded solely as SJS within our data, then the SCORTEN for these patients would be incorrect. The available data do not provide any information on the laboratory and physiological parameters before admission to intensive care, and certain prognostic factors of particular relevance to these patients, for example the percentage body surface area involvement, are not recorded in the database.

The conditions included within the ICNARC coding method [12], used to code the reasons for admission to ICU, are not fully comprehensive. However, as this is a hierarchical system, a method exists for coding any condition not specified in the coding method by completing the hierarchical coding to as much depth as possible and then recording the actual condition in the free text field. We identified 213 partially coded dermatological admissions, of which 161 could be fully classified using the information in the text field. For example, squamous cell carcinomas are not included in the coding method but can be recorded as 'surgical/dermatological/skin/tumour or malignancy' with the final condition of 'squamous cell carcinoma' in the free text field. It is, however, possible that some squamous cell carcinomas were miscoded as melanomas or basal cell carcinomas. Two squamous cell carcinomas recorded as basal cell carcinomas were identified and reclassified appropriately.

This study identifies patients with dermatological conditions requiring admission to intensive care. However, it provides no information on other patients with the same conditions, who require inpatient care, but are not severe enough to need intensive care or those who are not considered fit enough for intensive care. For example an elderly patient with cellulitis might die from sepsis, but not have been considered to be a candidate for intensive care. Conversely, more severe cases may have been referred directly to burns units, about which we have no data. A number of patients with infective conditions, TEN and related conditions, and scleroderma were transferred to other ICUs. It is likely that some of these patients were transferred to burns units where treatment outcomes may be better for necrotising fasciitis and TEN [21,26].

While our study considers specifically those patients whose primary or secondary reason for admission to intensive care was a dermatological condition, there is also considerable scope for patients with other conditions in intensive care to require dermatological support [1,2]. The CMP dataset has recently been revised to incorporate the new Critical Care Minimum Data Set [27], which includes days of dermatological support (defined as patients with major skin rashes, exfoliation or burns, use of multiple large trauma dressings, or use of complex dressings). Once sufficient data are available, it will be possible to examine all admissions to intensive care that require such dermatological support.

## Conclusion

This study highlights the importance of dermatological conditions in the ICU. Skin conditions necessitating ICU admission may be associated with severe physiological abnormalities, and such patients have higher mortality and longer intensive care stays than average for the UK adult ICU population. This study provides further evidence for the importance of skin failure as a distinct entity, comparable to other major organ system failures, with high mortality.

## Key messages

• Some patients with severe skin conditions require intensive care.

• These conditions include toxic epidermal necrolysis, Stevens–Johnson syndrome, necrotising fasciitis, exfoliative dermatitis, and cutaneous malignancies.

• ICU admission may be the result of the skin condition alone, e.g. for acute skin failure, or following complications of the condition or its treatment, e.g. following complex surgery for a malignant skin tumour.

• Patients with acute skin failure have higher mortality and longer intensive care stays than average for the UK adult ICU population.

## List of abbreviations

APACHE = Acute Physiology And Chronic Health Evaluation; CMP = Case Mix Programme; ICNARC = Intensive Care National Audit & Research Centre; ICU = intensive care unit; SJS = Stevens–Johnson syndrome; TEN = toxic epidermal necrolysis.

## Competing interests

The authors declare that they have no competing interests.

## Authors' contributions

SMCG and DAH conceived the study. SMCG, DAH, KMN and PSF agreed the analysis plan. DAH and CAW performed the analyses. SMCG and DAH drafted the manuscript. All authors contributed to interpretation of the results and critical revision of the manuscript and have seen and approved the final version.

## References

[B1] Green T, Manara AR, Park GR (1989). Dermatological conditions in the intensive care unit. Hosp Update.

[B2] Dunnill MGS, Handfield-Jones SE, Treacher D, McGibbon DH (1995). Dermatology in the intensive care unit. Br J Dermatol.

[B3] Irvine C (1991). 'Skin failure' – a real entity: discussion paper. J R Soc Med.

[B4] Inamadar AC, Palit A (2005). Acute skin failure: concept, causes, consequences and care. Indian J Dermatol Venereol Leprol.

[B5] Letko E, Papaliodis DN, Papaliodis GN, Daoud YJ, Ahmed AR, Foster CS (2005). Stevens–Johnson syndrome and toxic epidermal necrolysis: a review of the literature. Ann Allergy Asthma Immunol.

[B6] Roujeau JC (1994). The spectrum of Stevens–Johnson syndrome and toxic epidermal necrolysis: a clinical classification. J Invest Dermatol.

[B7] Hasham S, Matteucci P, Stanley PRW, Hart NB (2005). Necrotising fasciitis. BMJ.

[B8] Black N (1997). Developing high quality clinical databases. BMJ.

[B9] Kaufman DW (1994). Epidemiological approaches to the study of toxic epidermal necrolysis. J Invest Dermatol.

[B10] Rzany B, Mockenhaupt M, Baur S, Schröder W, Stocker U, Mueller J, Holländer N, Bruppacher R, Schöpf E (1996). Epidemiology of erythema exsudativum multiforme majus, Stevens–Johnson syndrome, and toxic epidermal necrolysis in Germany (1990–1992): structure and results of a population-based registry. J Clin Epidemiol.

[B11] Harrison DA, Brady AR, Rowan K (2004). Case mix, outcome and length of stay for admissions to adult, general critical care units in England, Wales and Northern Ireland: the Intensive Care National Audit & Research Centre Case Mix Programme Database. Crit Care.

[B12] Young JD, Goldfrad C, Rowan K (2001). Development and testing of a hierarchical method to code the reason for admission to intensive care units: the ICNARC Coding Method. Br J Anaesth.

[B13] Knaus WA, Draper EA, Wagner DP, Zimmerman JE (1985). APACHE II: a severity of disease classification system. Crit Care Med.

[B14] Harrison DA, Parry GJ, Carpenter JR, Short A, Rowan K A new risk prediction model for critical care: the Intensive Care National Audit & Research Centre (ICNARC) model. Crit Care Med.

[B15] Bastuji-Garin S, Fouchard N, Bertocchi M, Roujeau JC, Revuz J, Wolkenstein P (2000). SCORTEN: a severity-of-illness score for toxic epidermal necrolysis. J Invest Dermatol.

[B16] Hanley JA, McNeil BJ (1982). The meaning and use of the area under a receiver operating characteristic (ROC) curve. Radiology.

[B17] Harrison DA, D'Amico G, Singer M (2007). Case mix, outcome, and activity for admissions to UK critical care units with severe acute pancreatitis: a secondary analysis of the ICNARC Case Mix Programme Database. Crit Care.

[B18] Woodhead M, Welch CA, Harrison DA, Bellingan G, Ayres JG (2006). Community-acquired pneumonia on the intensive care unit: secondary analysis of 17,869 cases in the ICNARC Case Mix Programme Database. Crit Care.

[B19] Anaya DA, McMahon K, Nathens AB, Sullivan SR, Foy H, Bulger E (2005). Predictors of mortality and limb loss in necrotizing soft tissue infections. Arch Surg.

[B20] Endorf FW, Supple KG, Gamelli RL (2005). The evolving characteristics and care of necrotizing soft-tissue infections. Burns.

[B21] Faucher LD, Morris SE, Edelman LS, Saffle JR (2001). Burn center management of necrotizing soft-tissue surgical infections in unburned patients. Am J Surg.

[B22] Elliott DC, Kufera MA, Myers RAM (1996). Necrotizing soft tissue infections: risk factors for mortality and strategies for management. Ann Surg.

[B23] Guégan S, Bastuji-Garin S, Poszepczynska-Guigné E, Roujeau JC, Revuz J (2006). Performance of the SCORTEN during the first five days of hospitalization to predict the prognosis of epidermal necrolysis. J Invest Dermatol.

[B24] Cunningham JD, Silver L, Rudikoff D (2001). Necrotizing fasciitis: a plea for early diagnosis and treatment. Mt Sinai J Med.

[B25] Department of Health (2006). Our health, our care, our say: a new direction for community services.

[B26] Hettiaratchy S, Moloney D, Clarke J (2001). Patients with acute skin loss: are they best managed on a burns unit?. Ann R Coll Surg Engl.

[B27] UK Department of Health, Computing for Health Critical Care Minimum Data Set. http://www.dh.gov.uk/assetRoot/04/12/65/14/04126514.pdf.

[B28] Case Mix Programme participating units. http://www.icnarc.org/audit/cmp/participating-units.

